# Polymorphism-driven immune disruptions in Kawasaki disease across populations: decoding the role of T and B-cells

**DOI:** 10.3389/fimmu.2025.1640024

**Published:** 2025-10-03

**Authors:** Chu Zhang, Lu Wang, Qihong Fan, Yan Pan

**Affiliations:** The First Affiliated Hospital of Yangtze University, Jingzhou, China

**Keywords:** Kawasaki disease, T cell, B cell, coronary artery lesions, gene polymorphisms

## Abstract

Kawasaki disease (KD) is a self-limiting, systemic vasculitic syndrome of unknown etiology that primarily affects children under the age of five, with notably high incidence in Asian populations. Although initial treatment with high-dose intravenous immunoglobulin (IVIG) and aspirin can reduce acute symptoms of KD and the risk of coronary artery lesions (CALs), diagnosis remains challenging due to the absence of specific biomarkers and the incomplete understanding of disease pathogenesis, often resulting in misdiagnosis or delayed intervention. Genetic predisposition and immune dysregulation, particularly involving B-cell and T-cell pathways, have been implicated in KD susceptibility and the development of CAL. This review summarizes current evidence on immune-regulatory gene polymorphisms, with a focus on how T-cell and B-cell–related genetic variations may contribute to disease onset and vascular complications. These insights may help inform improved diagnostic accuracy—particularly for incomplete KD—and support personalized treatment strategies, such as corticosteroids or anti-TNF agents in genetically high-risk patients.

## 1 Introduction

Kawasaki disease (KD) is known for its significant geographic variation in incidence, with especially high rates observed in Asian populations, particularly in children under five years old (1). In Japan, the incidence of KD is notably high, with a reported rate of 359 per 100,000 children (2). Other regions in Northeast Asia, such as South Korea and Taiwan, also report higher incidence rates, with figures of 195 per 100,000 in South Korea and 60 per 100,000 in Taiwan (2). In China, studies show varying rates, with Shanghai reporting 107.3 per 100,000 in 2017 and Beijing 55.1 per 100,000 in 2004 (2, 3). However, the actual incidence in other parts of China remains uncertain because of the lack of a centralized national database (4), underscoring the need for more comprehensive national surveillance. In contrast, KD incidence in non-Asian regions is lower. The United States, for example, reports a rate of 18 to 25 per 100,000 children (5), while Canada reports 19.6 per 100,000 and Europe ranges from 4.5 to 9 per 100,000 children (2). These regional differences highlight the potential role of genetic predisposition and population-specific factors in KD susceptibility (6).

KD is a significant pediatric condition, presenting not only a medical challenge but also significant emotional and logistical burdens (4). KD often presents with persistent fever and irritability, which overlap with common infections and delay diagnosis, especially in incomplete cases lacking typical criteria (7). Early diagnosis of KD involves recognizing characteristic symptoms, such as persistent fever and conjunctival injection, and differentiating them from other febrile illnesses, particularly in the case of incomplete KD, where typical clinical signs may not be fully present (8). These diagnostic challenges are compounded by the potential for long-term cardiovascular complications, such as CAL. Although IVIG is the standard treatment, 10–20% of patients show resistance, increasing their risk of CAL (9). These complications may require long-term cardiovascular monitoring, placing significant psychological and economic strain on affected families (4). Even after defervescence, most children require serial echocardiographic follow-up and activity guidance; patients with coronary involvement often need prolonged antiplatelet therapy and cardiology visits, extending the burden into the convalescent phase (10). Therefore, refining early diagnosis and identifying patients at higher risk remain urgent priorities. In practice, early diagnosis means recognizing KD early in emergency and inpatient settings, applying standardized echocardiography-based evaluation, and distinguishing incomplete KD from common viral illnesses to avoid delayed IVIG administration.

Beyond the clinical challenges, KD also imposes significant psychological and economic burdens on families. Parents of children with KD often face considerable emotional distress as they navigate the uncertainties of their child’s illness and its long-term consequences (11). Studies have shown that parents experience higher levels of anxiety and depression during their child’s hospitalization and treatment for KD (11). Many families also face significant financial strain due to the high costs of IVIG therapy and long-term cardiovascular monitoring (12). For example, studies have shown that KD leads to substantial health care costs (13).

To address these clinical challenges, recent studies have increasingly focused on the genetic and immunological mechanisms underlying KD (14). KD’s immune dysregulation is marked by dual lymphocyte activation. Both T-cell hyperactivity and B-cell proliferation contribute to an immunoglobulin-driven immune response in KD pathophysiology, while excessive pro-inflammatory cytokine storms further exacerbate tissue damage (15, 16).

KD shares several immunopathogenic features with other immune-mediated diseases that present with fever and systemic inflammation. For instance, rheumatic fever, triggered by Group A streptococcal infection, involves T-cell activation and immune complex formation, leading to vascular inflammation, a feature also observed in KD (17). Similarly, systemic lupus erythematosus (SLE) and juvenile idiopathic arthritis (JIA) exhibit T-cell dysregulation and excessive cytokine production, leading to systemic inflammation and vascular damage (18), akin to the processes in KD. In addition, toxic shock syndrome (TSS) and measles both involve cytokine storms and exaggerated immune responses (19), presenting mechanisms that overlap with KD. These diseases highlight the role of immune dysregulation in driving vascular injury and systemic inflammation. Moreover, B-cell activation plays a central role in diseases like SLE (20), where immune complex formation and autoantibody production contribute to vascular damage, processes also seen in KD. These comparisons suggest that KD shares common immune dysregulation features with other diseases such as SLE, JIA, and rheumatic fever. Importantly, we can learn from these diseases’ immune responses, particularly how T-cell activation and B-cell-driven immune complexes contribute to vascular injury and inflammation. This knowledge may help guide the development of targeted therapies for KD, such as cytokine inhibitors and immune modulators, which are already used in diseases like SLE and rheumatic fever (21) and could be applied to KD.

Genetic polymorphisms in immune-related genes, including *ITPKC*, *FCGR2A*, and *CD40*, have been shown to modulate immune responses and IVIG resistance (22). Targeted therapeutic strategies in KD refer to treatments personalized according to such genetic risk profiles. For instance, polymorphisms in *ITPKC*, *FCGR2A*, and *CD40* can predict IVIG resistance, guiding the early use of adjunctive corticosteroids, anti-TNF agents (23–26), or calcineurin pathway modulation. In this review, “targeted therapy” specifically denotes such genotype-informed treatment choices, rather than a uniform regimen.

Epidemiological studies reveal distinct patterns of KD, including age stratification, gender predisposition, seasonal variation, geographic distribution, and familial clustering (27). These observations suggest that KD is influenced by a combination of genetic susceptibility and environmental factors, with higher prevalence in Asian populations (28). Additionally, emerging regulatory mechanisms, such as noncoding RNAs (ncRNAs), particularly microRNAs (miRNAs), long non-coding RNAs (lncRNAs), and circular RNAs (circRNAs), are involved in regulating immune responses and inflammation in KD (29). These factors may offer insight into the immune dysregulation and heterogeneity of KD across different populations.

In conclusion, timely recognition of incomplete KD and genotype-informed adjunctive therapies are important for reducing coronary complications and alleviating the long-term psychological and economic burdens for families.

In addition to exploring single-nucleotide polymorphisms (SNPs) associated with KD susceptibility and IVIG resistance, we also highlight their potential direct associations with CALs, the most severe complication of KD. To enhance conceptual clarity and better reflect the immunological basis of KD and its complication—CALs—we organized the discussion of SNPs based on their predominant involvement in either T-cell– or B-cell–mediated immune responses. Given its well-established mechanistic link to both KD pathogenesis and CAL development, we begin with *TNF* rs1800629 before moving to other T cell–related SNPs (*ORAI1*, *ITPKC*) and subsequently to B cell–related SNPs (*FCGR2A*, *CD40*, *BLK*).

## 2 T cell–related gene polymorphisms associated with KD susceptibility

T cell–related polymorphisms may contribute to KD pathogenesis and CAL development through cytokine dysregulation, calcium signaling, and NFAT-mediated immune activation.

### 2.1 *TNF* rs1800629

Located on chromosome 6p21.1–21.3, within the highly polymorphic major histocompatibility complex (MHC) region, the tumor necrosis factor (*TNF*) gene contributes to immune regulation (30). Its product, TNF-α, is a key cytokine involved in immune regulation and pathogen defense. Clinical evidence suggests that TNF-α antagonists significantly improve therapeutic resistance in KD management when compared to standard IVIG therapy alone (31).

The rs1800629 polymorphism (−308G>A) resides in the promoter region of the *TNF* gene and has been associated with elevated TNF-α expression (30), particularly in individuals carrying the AA and AG genotypes (32). During the acute phase of KD, TNF-α promotes vascular endothelial activation, upregulates Intercellular Adhesion Molecule-1 (ICAM-1) and Monocyte Chemoattractant Protein-1 (MCP-1) expression, and contributes to leukocyte recruitment and vascular inflammation (25). Synergistic interactions between TNF-α and IL-1β amplify inflammatory cascades, leading to endothelial dysfunction and structural damage (33). These molecular events establish a self-sustaining cycle of oxidative stress and inflammatory activation within arterial walls, thereby contributing to the hallmark clinical features of KD (34). These processes enhance endothelial permeability and induce pro-thrombotic states, and could potentially contribute to cardiovascular complications (35). TNF-α also activates signaling pathways that promote vascular smooth muscle cell proliferation and extracellular matrix deposition, resulting in arterial abnormalities such as stenosis to aneurysm formation (25, 36).

Notably, TNF-α has been shown to induce endothelin-1 (ET-1) production in mononuclear cells through paracrine signaling (37). ET-1, a vasoconstrictor secreted by vascular endothelial cells (38), acts via the endothelin A receptor to promote vasoconstriction and vascular remodeling, which may contribute to vascular injury in KD (39).

Functional studies confirm that the A allele of rs1800629 increases TNF-α, IL-8, and IL-1β production, and enhances neutrophil migration (40). Human cellular models reveal that A/G genotypes exhibit higher TNF-α expression upon lipopolysaccharide stimulation (41), and the A allele of the TNF-α-308 genotype has been implicated in the pathogenesis of certain autoimmune diseases, such as psoriasis and rheumatoid arthritis (42). Moreover, TNF-α influences lipid metabolism, insulin resistance, and endothelial function, contributing to coronary heart disease risk, particularly in individuals with type 2 diabetes (43, 44).

In KD, while no significant difference in rs1800629 genotype frequencies was found between affected and healthy children in the Chinese Han population, the A allele was significantly enriched among IVIG-resistant patients (45). This allele frequency was also comparable to that in Japanese and Caucasian populations (45). A meta-analysis across predominantly East Asian populations found no consistent association between this SNP and overall KD susceptibility (46). In contrast, studies in Iranian patients reported that the GG haplotype of rs1800629 was significantly associated with increased KD risk (47).

### 2.2 *ORAI1* rs3741596

Located on chromosome 12q24.31 (48), the calcium release-activated calcium modulator 1(*ORAI1*) gene encodes the hexameric ORAI1 protein, which forms calcium release-activated calcium (CRAC) channels. These channels are vital mediators of store-operated calcium entry (SOCE) in immune cells (49, 50). ORAI1, together with stromal interaction molecule 1 (STIM1), forms a calcium influx mechanism essential for T cell activation (51). Upon T cell receptor (TCR) engagement, inositol 1,4,5-trisphosphate (IP_3_)-mediated endoplasmic reticulum (ER) calcium depletion triggers conformational changes in STIM1, which in turn activate ORAI1 channels on the plasma membrane, permitting extracellular Ca²^+^ influx (52, 53). This rise in intracellular Ca²^+^ activates the calcineurin-nuclear factor of activated T-cells (NFAT) signaling cascade, which promotes transcription of pro-inflammatory cytokines and contributes to T-cell–mediated immune activation (54).

The non-synonymous mutation at the rs3741596 locus substitutes glycine for serine at position 218 of the ORAI1 protein (55), which may alter the structure of the CRAC channel. This alteration may enhance channel activity, increase extracellular calcium ion influx, and consequently induce intracellular calcium overload (56). Additionally, this mutation might modulate the interaction between ORAI1 and associated proteins such as STIM1, a calcium sensor located on the endoplasmic reticulum (57). Under normal conditions, depletion of the endoplasmic reticulum calcium store triggers STIM1 to activate the ORAI1 channel, facilitating calcium influx (58). The rs3741596 mutation could strengthen the binding affinity between ORAI1 and STIM1, leading to dysregulation of calcium influx (59). A case report from Taiwan indicated that this mutation could lead to constitutive Ca²^+^ entry into immune cells and sustained inflammatory signaling, thereby promoting resistance to IVIG, glucocorticoids, and infliximab (50, 60). These functional changes highlight ORAI1’s pivotal role in T cell activation and calcium signaling, which may contribute to immune dysregulation and vascular inflammation in Kawasaki disease (61).

In Chinese children, the rs3741596 G allele was significantly associated with KD and CALs (62). Japanese studies consistently identified the G allele as a genetic risk factor (63). Large-scale Japanese studies—including discovery, replication, and meta-analyses—further confirmed the G allele as a significant genetic risk factor for KD (55). Functionally, *ORAI1* variants may alter calcium influx and downstream NFAT signaling, contributing to immune dysregulation and vascular inflammation during KD pathogenesis (55).

### 2.3 ITPKC rs28493229

Located on chromosome 19q13.2 (64), the inositol-trisphosphate 3-kinase C (*ITPKC*) gene encodes the enzyme inositol 1,4,5-trisphosphate 3-kinase C, which phosphorylates Inositol 1,4,5-trisphosphate (IP_3_) —a second messenger that mobilizes intracellular calcium—and thereby suppresses Ca²^+^/NFAT signaling pathways (6). Although its precise involvement in store-operated calcium (SOC) channel regulation remains uncertain, variations in *ITPKC* expression levels may influence IP_3_ metabolism and consequently affect SOC channel activation (65).

Onouchi et al. (65) identified the *ITPKC* rs28493229 polymorphism as functionally significant, demonstrating its association with elevated IVIG resistance risk in KD patients. Their cellular investigations revealed the C allele at this locus reduces mRNA splicing efficiency by approximately 30%, leading to diminished gene expression and hyperactivation of NFAT signaling in T lymphocytes (65). This molecular mechanism may elucidate the immune dysregulation characteristic of KD pathophysiology (66). Histopathological studies in acute KD demonstrate coronary arterial T cell infiltration and elevated IL-2 expression, consistent with enhanced T cell activation (67). In genetically engineered animal models, *ITPKC*-deficient mice challenged with lipopolysaccharide exhibit increased IL-1β production and more severe disease phenotypes compared to wild-type controls (68). These findings suggest that microbial triggers may amplify T-cell–driven inflammation in genetically susceptible individuals, further compromising vascular endothelial integrity (69).

Geographical variation in the association of rs28493229 in the *ITPKC* gene with KD has been observed across populations (6). In Taiwanese, results are inconsistent—some studies found no link with KD or CALs, while others observed C allele associations with CAL risk (70, 71). No significant associations were found in Mainland Chinese cohorts (72). Additionally, large-scale genome-wide association studies (GWAS) involving Japanese and Caucasian cohorts demonstrated that the C allele of rs28493229 was significantly associated with both KD susceptibility and CAL development (65).

These findings suggest that genetic variations in *ITPKC* may modulate T-cell activation and predispose individuals to CAL development by sustaining vascular inflammation through dysregulated Ca²^+^/NFAT signaling.

## 3 B cell–related gene polymorphisms associated with KD susceptibility

These polymorphisms may influence B-cell activity via multiple mechanisms, including altered Fc receptor affinity, enhanced B–T co-stimulation, and class-switch recombination under inflammatory conditions.

### 3.1 *FCGR2A* rs1801274

Located on chromosome 1q23.3 (73), the Fc gamma receptor IIA (*FCGR2A)* gene has emerged as a key contributor to KD susceptibility across diverse ethnic groups (74). It encodes Fc gamma receptors (FcγRs), which mediate immunoglobulin G (IgG) binding and regulate immune responses through multiple mechanisms (75). FcγRs are widely expressed on the membranes of various immune cells, including dendritic cells, macrophages, NK cells, neutrophils, and B lymphocytes (76). The binding of immune complexes to FcγRIIA initiates intracellular signaling cascades, facilitating internalization and activation of downstream pathways (77). This engagement enhances phagocytic activity and promotes pro-inflammatory cytokine production through signal transduction pathways (78). Disease-associated polymorphisms in Fc receptors may influence autoimmune pathogenesis by altering IgG-binding affinities (79).

The nonsynonymous SNP rs1801274 (A>G) in *FCGR2A* causes a histidine-to-arginine substitution at position 131, altering receptor affinity for Immunoglobulin G subclass 2 (IgG2), a subclass of immunoglobulin G specialized in responses to polysaccharide antigens (80). A-allele homozygotes exhibit stronger IgG2 binding and enhanced phagocytosis (81), which may prolong immune complex retention and trigger persistent immune activation, contributing to vascular injury and CALs in KD.

In B cells, FcγRIIB transmits inhibitory signals by recruiting SH2 domain–containing inositol 5-phosphatase 1 (SHIP-1) and Src homology 2 domain–containing protein tyrosine phosphatase 1 (SHP-1)-two phosphatases known to downregulate immune signaling (82). In contrast, FcγRIIA promotes B-cell activation through distinct signaling cascades (83–85). This signaling divergence may modulate how B cells respond to immune complexes. In the context of KD, a shift toward FcγRIIA-dominant signaling may enhance B-cell activation, thereby contributing to or exacerbating vascular inflammation and coronary artery involvement. Furthermore, elevated FcγRIIA/IIB mRNA expression ratios have been observed in KD patients with CALs (86), and the G allele has been linked to enhanced endothelial vasodilation and nitric oxide production under stimulation (81). The rs1801274 variant may disrupt this balance, impairing co-inhibition and resulting in heightened B cell activation, autoantibody production, and inflammation.

A growing body of evidence has linked rs1801274 to KD risk and CAL development (87). However, Ethnic-specific studies report inconsistent associations, suggesting population-based variability in *FCGR2A*-related susceptibility. For example, a Chinese study demonstrated that the AG genotype or G allele of rs1801274 was significantly associated with an increased risk of KD (88). Meta-analyses have confirmed significant associations between the A allele of rs1801274 and increased KD susceptibility, particularly among Asian populations (81). A Korean clinical study identified *FCGR2A* as a significant risk factor for KD, particularly in male infants under 1 year of age (89). In contrast, studies conducted in a Greek population revealed no significant association between *FCGR2A* and KD, underscoring the role of ethnic heterogeneity in genetic predisposition (90).

### 3.2 *CD40* rs1535045

Located on chromosome 20q13.12, the *CD40* gene encodes a 50 kDa type I transmembrane glycoprotein that belongs to the tumor necrosis factor receptor superfamily (91). CD40 is primarily expressed on B lymphocytes and mediates cellular activation via CD40L-induced multimerization, initiating tumor necrosis factor receptor-associated factor (TRAF)*-*mediated signal transduction pathways essential for B-cell proliferation and antibody production (14).

Notably, the TT genotype of the rs1535045 polymorphism has been linked to an increased susceptibility to KD, possibly due to dysregulated immune responses that contribute to vascular wall abnormalities (91). The CD40/CD40L axis also contributes to cardiovascular disorders, with elevated CD40L expression observed on CD4^+^ T cells and platelets during the acute phases of KD, coinciding with increased serum soluble CD40L concentrations (92). This signaling cascade facilitates B-cell activation and antibody production, and when dysregulated, it may provoke systemic vasculitis via B-cell hyperactivation, antibody overproduction, and the release of inflammatory cytokines, such as TNF-α, IL-6, and IL-10 (93).

Previous studies have also shown that the rs1535045 T allele is more prevalent than the C allele in individuals with coronary artery disease (94). Clinical studies in Chinese (95) and Taiwanese (96) populations identified the TT genotype of *CD40* rs1535045 as a risk factor for KD, with the Chinese study also noting no association with CALs. A meta-analysis including North Indian populations further supported the risk association of the T allele (93). However, the Indian cohort study did not observe a statistically significant correlation between rs1535045 and KD risk, although elevated CD40 expression was noted in B cells from KD patients with coronary aneurysms (93), suggesting possible post-transcriptional involvement.

### 3.3 *BLK* rs2736340

Located on chromosome 8p23.1 (97), the B lymphocyte tyrosine kinase (*BLK*) gene, encodes a non-receptor tyrosine kinase predominantly expressed in B cells, where it plays a crucial role in B cell maturation, B cell receptor (BCR)-mediated signaling, and nuclear factor kappa-light-chain-enhancer of activated B cells (NF-κB) pathway modulation (98–100), a key regulator of immune and inflammatory responses. BLK regulates early B-cell development and antigen-driven activation through coordination with B-cell activating factor (BAFF), a cytokine belonging to the TNF superfamily (101). This interaction promotes B-cell survival and amplifies immune signaling via BCR and NF-κB pathways (101–103). In addition to antibody secretion, B cells also exert regulatory roles by producing anti-inflammatory cytokines and interacting with T cells (104). Dysfunction in this regulatory axis may contribute to immune dysregulation in KD (60). Notably, the *BLK* rs2736340 polymorphism demonstrates consistent association with KD susceptibility across Asian populations, particularly in Taiwanese, Japanese, and Korean cohorts (105). Korean studies demonstrated diminished BLK expression in B cell lineages from KD patients carrying the risk-associated T allele, with parallel findings in purified acute-phase B lymphocytes (106).

Mechanistically, the T allele impairs BCR signaling, as evidenced by attenuated extracellular signal–regulated kinase (ERK) phosphorylation, a key downstream mediator that promotes B cell proliferation and survival in the BCR pathway (106, 107). This impaired signaling may result in diminished antibody production and weakened anti-inflammatory capacity (108). Furthermore, reduced BLK function may hamper regulatory B cell activation, leading to exaggerated T cell proliferation and increased Th1-type responses, thereby aggravating vascular inflammation (109).

Population-level analyses in Korean and European cohorts establish significant correlations between the rs2736340 T allele and decreased BLK expression in circulating B lymphocytes during acute Kawasaki episodes, indicating functional alterations in lymphocyte behavior relevant to disease pathogenesis (106). Chinese clinical investigations corroborated these findings, demonstrating elevated TT genotype and T allele frequencies at rs2736340 in incomplete KD cases compared to controls, which suggests potential genetic predisposition patterns (110). Additionally, a genome-wide association study in a Han Chinese population reported that the C allele at rs2736340 may confer protection against KD susceptibility (111).

Taken together, *BLK* polymorphisms may compromise regulatory B-cell function, leading to enhanced T-cell activation and vascular injury, ultimately contributing to the formation of CALs.

## 4 Discussion

### 4.1 Toward a unified immune mechanism for KD pathogenesis

The preceding sections analyzed how individual genetic polymorphisms affect B- and T-cell signaling. Here, we synthesize those findings to elucidate a unified immunopathogenic model for KD.

This study investigated the association between KD and genetic polymorphisms related to T- and B-cell-mediated immunity, with a particular focus on the roles of *ITPKC*, *ORAI1*, and *TNF* in T-cell immunity, and *FCGR2A*, *CD40*, and *BLK* in B-cell immunity. To facilitate cross-regional comparison, **Table 1** organizes study results by country/region, clearly indicating observed associations or the absence thereof, as well as the sample size of each study. It synthesizes recent studies to elucidate how these polymorphisms influence immune mechanisms in KD and CALs, suggesting potential clinical utility of these genetic factors. This review provides a balanced synthesis of T- and B-cell immunity in KD pathogenesis.

**Table 1:** Summary of genetic association studies on six immune-regulatory SNPs in Kawasaki disease across different populations.

Gene (SNP)	Country/Region	Ethnicity	Study design	Sample size (Cases/Controls)	Associated allele	Risk/Protection	Clinical relevance (e.g., CALs)	Reference
*TNF* (rs1800629)	China (Mainland)	Han Chinese	Case-control	96 / 160	A	Risk (IVIG resistance)	IVIG resistance	(45)
*TNF* (rs1800629)	Asia	Predominantly East Asian	meta-analysis	NA	NA	NA	No association	(46)
*TNF* (rs1800629)	Iran	Iranian	Case-control	55/140	GG haplotype	Risk	KD susceptibility	(47)
*ORAI1* (rs3741596)	China (Mainland)	Han Chinese	Case-control	46/25	G	Risk	KD susceptibility, CALs (G allele)	(62)
*ORAI1* (rs3741596)	Japan	Japanese	Case–control discovery + replication + meta-analysis	729/1,315 (discovery); 1,813/1,097 (replication); 2,542/2,412 (meta)	G	Risk	KD susceptibility; Ca²^+^/NFAT pathway implicated	(55)
*ITPKC* (rs28493229)	Taiwan	Han Chinese	Case-control + Family-based (TDT)	385 / 1,158 (plus 184 trios)	NA	NA	No association with KD or CALs	(70)
*ITPKC* (rs28493229)	Taiwan	Han Chinese	Case-control + Meta-analysis	341 / 1,190 (plus meta)	C	Risk	CALs	(71)
*ITPKC* (rs28493229)	China (Mainland)	Han Chinese	Case-control	206 / 285	NA	NA	No association with KD or CALs	(72)
*ITPKC* (rs28493229)	Japan / USA	Asian + Caucasian	GWAS	Not specified	C	Risk	KD susceptibility & CALs	(65)
*BLK* (rs2736340)	China (Mainland)	Han Chinese	Case-control	184/203	T	Risk	Incomplete KD susceptibility	(110)
*FCGR2A* (rs1801274)	China (Mainland)	Han Chinese	Case-control	35/25	A, G	Risk	KD susceptibility (A allele), CALs (G allele)	(88)
*FCGR2A* (rs1801274)	Korea	Korean	Case-control	1011/4533	NA	Risk	KD susceptibility (only in males <1 yr)	(89)
*FCGR2A* (rs1801274)	China (Mainland)	Han Chinese	Case-control	428/493	A	Risk	KD susceptibility	(81)
*FCGR2A* (rs1801274)	Greece	Greek	Case-control	47/50	NA	NA	No association	(90)
*FCGR2A* (rs1801274)	Multi-country	Mixed (mostly Asian & Caucasian)	Meta-analysis	3,673 / 14,226 + TDT	A	Risk	KD susceptibility	(81)
*CD40* (rs1535045)	India	North Indian	Case-control& meta-analysis	51/41	T	Risk	KD susceptibility (meta-analysis)	(93)
*CD40* (rs1535045)	China (Mainland)	Han Chinese	Case-control	184 / 206	T	Risk	KD susceptibility; no CALs association	(95)
*CD40* (rs1535045)	Taiwan	Han Chinese	Case-control	381 / 569	NA	NA	KD susceptibility	(96)
*BLK* (rs2736340)	China (Mainland)	Han Chinese	Case-control	184/203	T	Risk	Incomplete KD susceptibility	(110)
*BLK* (rs2736340)	China (Mainland)	Han Chinese	GWAS	428/493	C	Protection	reduce susceptibility	(111)
*BLK* (rs2736340)	Taiwan / Japan / Korea (Asia)	Han Chinese / East Asian	GWAS replication & meta-analysis	2,539 / 7,021	T	Risk	KD susceptibility	(106)
*BLK* (rs2736340)	Europe / Western populations	Caucasian	GWAS replication & meta-analysis	405 / 6,252	T	Risk	KD susceptibility	(106)

SNP, single nucleotide polymorphism; CALs, coronary artery lesions; IVIG, intravenous immunoglobulin; NA, not available; TDT, Transmission Disequilibrium Test.

(page set in landscape orientation).

A key strength of this review lies in its emphasis on T- and B-cell immunoregulatory genes. While T-cell contributions to KD have been extensively studied, the role of B cells has often been neglected. However, given B cells’ critical involvement in immune responses and their influence on vasculitides and immune regulation, studying B-cell immunity becomes essential. This review examines how *FCGR2A*, *CD40*, and *BLK* polymorphisms contribute to immune dysregulation in KD.

Genetic polymorphisms in T- and B-cell regulatory genes have been proposed to form two converging immunopathogenic pathways (112) that may contribute to KD susceptibility and CAL formation. In the T-cell axis, *ITPKC* rs28493229 impairs IP_3_ production, while *ORAI1* rs3741596 has been reported to enhance calcium influx via the STIM1–ORAI1 axis (50)—both converge on sustained Ca²^+^/NFAT activation, elevating IL-2, IFN-γ, and pro-inflammatory cytokines. Meanwhile, *TNF* rs1800629 has been associated with increased TNF-α transcription (30), which may exacerbate systemic inflammation. In the B-cell arm, *FCGR2A* rs1801274 (A>G) enhances IgG2 binding and immune complex retention, promoting complement-mediated vascular inflammation (90, 113); *CD40* rs1535045 may upregulate T–B co-stimulation, and its variation may contribute to enhanced immunoglobulin class switching—i.e., the process by which B cells switch from producing IgM to other antibody isotypes such as IgG1 or IgG3—under pro-inflammatory conditions involving cytokines such as TNF-α and IL-6^+^ (114, 115); *BLK* rs2736340 has been associated with reduced BCR signaling, which may alter downstream immune regulation, including potential effects on γδT-cell activation and neutrophil responses (97, 116).

**Figure 1:**
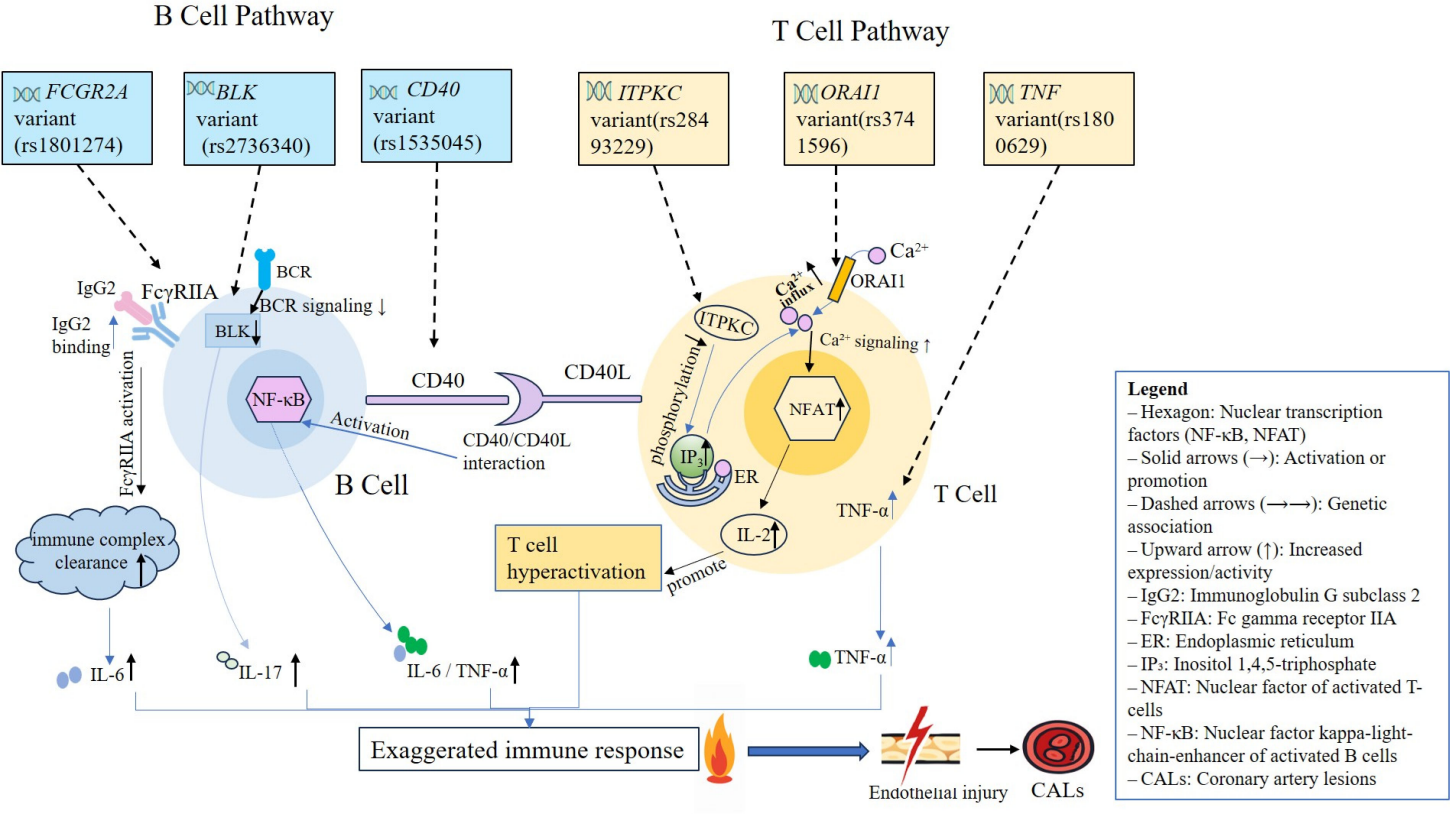
Proposed mechanisms of KD susceptibility-associated variants in B and T cells. This integrative model illustrates how six immune-regulatory SNPs disrupt T- and B-cell signaling pathways and converge on shared inflammatory cascades involving NFAT and NF-κB activation. The figure visually connects gene-level variation to immune dysregulation and vascular injury, thereby highlighting a unified pathophysiological mechanism underlying KD susceptibility and coronary complications. This schematic illustrates the putative cellular and molecular mechanisms through which six KD-associated genetic variants may contribute to immune dysregulation. In T cells (right), *TNF* variant rs1800629 elevates TNF-α expression, amplifying inflammatory responses. The *ORAI1* variant rs3741596 alters Ca²^+^ influx, promoting cytokine production. The *ITPKC* variant rs28493229 reduces IP_3_ phosphorylation, resulting in elevated NFAT and IL-2 levels, leading to T cell hyperactivation. In B cells (left), *BLK* variant rs2736340 leads to suppressed BCR signaling and upregulation of IL-17, contributing to Th17-related inflammation. *CD40* variant rs1535045 enhances NF-κB activation via CD40-CD40L interaction, promoting IL-6 and TNF-αproduction. *FCGR2A* variant rs1801274 increases IgG2 binding and FcγRIIA activation, modulates immune-complex handling and downstream inflammatory signaling. These genetic variants may cooperatively contribute to aberrant immune activation and CALs in KD.

These pathways activate common downstream effectors (NFAT, NF-κB), leading to elevated TNF-α, IL-1β, and IL-17 levels, endothelial dysfunction, leukocyte adhesion, and vascular remodeling. Taken together, current evidence suggests that these T- and B-cell-related SNPs converge on shared inflammatory pathways (e.g., NFAT and NF-κB), which may contribute to a lymphocyte-driven cytokine storm and immune-mediated vascular injury in KD. These immune disturbances may ultimately manifest as persistent fever, mucocutaneous inflammation, and coronary artery complications—the cardinal features of KD (117). This mechanistic integration constitutes a proposed unified immunopathogenic model of KD, as visually summarized in **Figure 1**. Ethnic-specific allele frequencies (e.g., East Asian > Caucasian) further modulate the impact of these genes, explaining geographic gradients in KD incidence (118). This integrative model underscores the importance of genotype-based immune targeting in KD. These immunopathogenic pathways are visually summarized in **Figure 1**, illustrating how T- and B-cell–related SNPs cooperatively contribute to immune dysregulation and vascular injury in Kawasaki disease.

### 4.2 Ethnic-specific genetic variations and global patterns

As summarized in **Table 1**, studies on six KD-associated SNPs reveal distinct ethnic-specific patterns. *ORAI1* rs3741596 displays reproducible associations in Chinese and Japanese groups. In contrast, *ITPKC* rs28493229 and *TNF* rs1800629 show variable associations across regions, particularly in relation to IVIG response and coronary complications. *FCGR2A* rs1801274, *CD40* rs1535045, and *BLK* rs2736340 show consistent associations with KD in Han Chinese and Taiwanese populations, while findings from Greek or Indian cohorts are inconsistent or null. *BLK* rs2736340 presents as a risk allele across East Asian and Western samples, though data from non-Asian populations remain limited.

These differences reflect the impact of population-specific genetic backgrounds and emphasize the need for large-scale, ethnically diverse replication studies. Discrepancies may result from variation in allele frequency, sample size, or study design. Future research should employ harmonized protocols and multi-ethnic cohorts to clarify these associations.

These population-specific differences in risk allele frequencies raise the possibility that East Asian populations may carry a higher cumulative genetic burden for KD-associated SNPs. This elevated burden has been hypothesized to partly explain the markedly higher incidence of KD observed in countries such as Japan, Korea, and China, compared to Western populations (2, 111).

### 4.3 Implications for personalized treatment

Another highlight of this study is the discussion of gene polymorphism-based personalized medicine for KD treatment. By analyzing genes like *FCGR2A*, *CD40*, and *BLK*, we demonstrate that genetic information could guide treatment decisions. Understanding genotypic variations in immune responses allows clinicians to develop more precise treatment plans, improving patient outcomes and minimizing CAL damage.

While this review discusses the clinical implications of individual SNPs, it is important to consider the possibility of gene–gene interactions. For example, *FCGR2A* rs1801274 enhances IgG2 binding and promotes immune complex formation (81, 119), whereas *BLK* rs2736340 reduces B-cell receptor signaling (106) and may dampen antibody production. The presence of both variants in the same individual raises the question of whether one effect may modulate or even counteract the other. Similarly, *ITPKC* rs28493229 and *ORAI1* rs3741596 both influence calcium–NFAT signaling (6, 54), and their combined effects could produce additive or nonlinear immune responses. These examples suggest that the functional significance of a single SNP may depend on the broader genotypic context, an area that remains underexplored in KD research. Future precision medicine strategies may benefit from integrating multi-locus profiles or epistatic (gene–gene interaction) modeling to better stratify patients and personalize treatment.

Recent studies underscore the promise of applying genetic polymorphism profiles to guide individualized therapy in Kawasaki disease (120). For instance, carriers of the *FCGR2A* rs1801274 A allele—linked to heightened immune complex formation due to enhanced IgG binding—may benefit from early adjunctive anti-inflammatory interventions, such as corticosteroids or anti-TNF-α agents, to mitigate the risk of CAL (81). Likewise, patients with the *ITPKC* rs28493229 C allele, which impairs Ca²^+^/NFAT signaling (60) and contributes to IVIG resistance (65), may respond more favorably to calcineurin inhibitors (e.g., cyclosporine A) or IL-1 receptor antagonists as part of initial treatment. In addition, *ORAI1* gain-of-function variants that amplify calcium influx in T cells have been implicated in overactive immune responses to guide individualized therapy (69). *ORAI1* gain-of-function variants, beyond guiding medication choices, may also support early risk stratification (7). Integrating genotyping into diagnostic workflows could help identify patients with high-risk immune signatures before treatment initiation—allowing clinicians to escalate therapy proactively in those at greater risk for IVIG resistance or severe cardiovascular outcomes (63). To realize these clinical applications, future research must extend beyond association studies to include large-scale, prospective trials that integrate genotyping with clinical monitoring. Such studies would help map genotype–phenotype–outcome relationships and support the development of risk-based treatment algorithms (121). Ultimately, this approach could lay the foundation for precision medicine in KD—improving outcomes while minimizing unnecessary interventions. To validate these genotype-guided strategies and support the clinical implementation of personalized medicine in KD, large-scale randomized controlled trials (RCTs) are urgently needed. Collectively, integrating genotypic profiling into KD management could facilitate earlier diagnosis, personalized treatment, and improved long-term cardiovascular outcomes.

Beyond the genes analyzed in this review, genome-wide association studies in non-Asian populations have identified additional susceptibility loci relevant to Kawasaki disease. Notably, *SMAD3* and *TGFB2*, both involved in the TGF-β signaling pathway, have been associated with increased risk of coronary artery complications in European-descended cohorts (122). Robust associations are mainly seen in East Asians (68), possibly reflecting regional prevalence and genetic architecture. Several hypotheses have been proposed to explain this Asia-predominant trend, including possible founder effects, region-specific infectious or environmental triggers, or population-level genetic bottlenecks (118, 123). Founder effects occur when a small group from a larger population establishes a new population, leading to reduced genetic diversity (63, 124). This limited genetic variation may result in certain genetic traits being more prevalent within that population. In KD, this has been hypothesized to contribute to higher frequencies of specific genetic variants, influencing disease susceptibility. Genetic bottlenecks happen when a population undergoes a dramatic reduction in size, causing the loss of genetic diversity (125). This reduction can cause certain alleles to become more common and might partly explain observed disparities in KD susceptibility between East Asian and non-Asian groups (126). These findings highlight the importance of vascular remodeling and immune regulation pathways in KD beyond classical T/B-cell mechanisms. Although these genes are not the primary focus of the current review, their identification in multi-ethnic studies underscores the need for expanded, population-specific genetic research, which could further refine our understanding of KD pathogenesis and its clinical heterogeneity across geographic regions.

### 4.4 Future directions and limitations

Limitations of the current study include the geographical bias of most research samples, which predominantly come from Asian populations. The generalizability of these findings across other ethnic groups remains uncertain. Moreover, while the selected SNPs—*TNF*, *ORAI1*, *ITPKC*, *FCGR2A*, *CD40*, and *BLK*—have been frequently reported in the KD literature, the strength of supporting evidence varies across loci. Some variants, such as *FCGR2A* rs1801274 and *ITPKC* rs28493229, have been validated by multiple studies in Japanese, Taiwanese, and Chinese populations. In contrast, others like *ORAI1* rs3741596 or *BLK* rs2736340 have more limited replication and are supported primarily by single-center studies. Therefore, their representativeness and robustness require further validation through high-powered, multi-ethnic replication studies and meta-analyses. Future studies should include diverse populations to verify the associations between gene polymorphisms and KD susceptibility. Additionally, there is a need for further investigation into how gene polymorphisms influence specific clinical phenotypes, such as CAL and cardiovascular complications. Experimental studies, including genetically engineered animal models and cellular research, could provide deeper insights into the functions of these genes in immune responses.

While this review highlights the significance of T- and B-cell immunoregulatory gene polymorphisms in KD susceptibility, translating these findings into practical clinical applications remains a significant challenge. The relationship between gene polymorphisms and clinical treatment strategies has not been systematically validated, particularly in terms of leveraging gene polymorphisms to optimize therapeutic approaches.

Translating these genetic insights into clinical practice remains a key challenge. While variants such as *FCGR2A* rs1801274 and *ITPKC* rs28493229 hold promise for guiding therapy selection or predicting IVIG resistance, prospective pharmacogenomic studies are needed to validate these approaches and define genotype-informed treatment algorithms.

KD patients who develop coronary complications may require long-term cardiovascular monitoring, antiplatelet therapy, or even surgical intervention (127). These long-term consequences not only impose a psychological and financial burden on families but also raise the risk of adult-onset cardiovascular disease (128).

Beyond these genetic considerations, environmental factors may also play a role in KD susceptibility. Epidemiological observations—such as seasonal fluctuations, geographic clustering, and temporal outbreaks—suggest that infections, pollutants, or allergens might act as environmental triggers in genetically predisposed individuals (129, 130). However, the interaction between genetic variants, particularly those involved in T- and B-cell regulation, and environmental exposures remains poorly characterized. Integrating environmental data into genetic association studies—such as through gene–environment interaction models or exposome-wide analyses (6, 131)—could provide deeper insights into KD pathogenesis. Addressing this gap would align the current review more closely with the broader research aim of understanding how environmental and genetic factors together influence autoimmune and autoinflammatory disorders.

Overall, this review offers a perspective on integrating immunogenetics into KD research by investigating the role of genetic polymorphisms in T- and B-cell-related immunity regarding KD susceptibility, and proposes promising directions for future studies. Although constrained by limited sample diversity, insufficient genotype-phenotype correlations, and unvalidated mechanisms, it establishes a framework for personalized KD therapeutics and immunological investigation. Further experimental validation and multiethnic clinical studies are warranted to translate these findings into improved diagnostic, therapeutic, and preventive approaches.
